# Clinical correlates of mathematical modeling of cortical spreading depression: Single‐cases study

**DOI:** 10.1002/brb3.1387

**Published:** 2019-09-10

**Authors:** Julia M. Kroos, Marina de Tommaso, Sebastiano Stramaglia, Eleonora Vecchio, Nicola Burdi, Luca Gerardo‐Giorda

**Affiliations:** ^1^ Basque Center for Applied Mathematics Bilbao Spain; ^2^ Applied Neurophysiology and Pain Unit, SMBNOS Department Bari Aldo Moro University Bari Italy; ^3^ Center of Innovative Technologies for Signal Detection and Processing TIRES, Physic Department Bari Aldo Moro University Bari Italy; ^4^ INFN Bari Italy; ^5^ Department of Radiology‐Neuroradiology Santissima Annunziata Hospital Taranto Italy

**Keywords:** cortical spreading depression, diffusion tensor imaging, finite element method, magnet resonance imaging, migraine with aura, patient‐specific mathematical model

## Abstract

**Introduction:**

Considerable connections between migraine with aura and cortical spreading depression (CSD), a depolarization wave originating in the visual cortex and traveling toward the frontal lobe, lead to the hypothesis that CSD is underlying migraine aura. The highly individual and complex characteristics of the brain cortex suggest that the geometry might impact the propagation of cortical spreading depression.

**Methods:**

In a single‐case study, we simulated the CSD propagation for five migraine with aura patients, matching their symptoms during a migraine attack to the CSD wavefront propagation. This CSD wavefront was simulated on a patient‐specific triangulated cortical mesh obtained from individual MRI imaging and personalized diffusivity tensors derived locally from diffusion tensor imaging data.

**Results:**

The CSD wave propagation was simulated on both hemispheres, despite in all but one patient the symptoms were attributable to one hemisphere. The CSD wave diffused with a large wavefront toward somatosensory and prefrontal regions, devoted to pain processing.

**Discussion:**

This case‐control study suggests that the cortical geometry may contribute to the modality of CSD evolution and partly to clinical expression of aura symptoms. The simulated CSD is a large and diffuse phenomenon, possibly capable to activate trigeminal nociceptors and to involve cortical areas devoted to pain processing.

## Graphical abstract

The arrival time (activation time) of the cortical spreading depression wave in the left (left side) and right (right side) hemisphere in one patient reporting visual and sensory migraine aura symptoms.

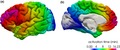

## 1 INTRODUCTION

The 2016 Global Burden of Disease study (GBD, 2019) reports that among neurological disorders, migraine is the second highest cause of years lost due to disability worldwide. One‐third of the migraine patients additionally experience a migraine aura preceding the typical headache. According to the current classification, typical migraine aura consists of visual, sensory, and speech disturbances, each reversible within 60 min (IHS, 2018). Since the pivotal study of Hadjikhani et al. (2001), CSD is considered to be the physiological substrate of the migraine aura that causes spreading of a self‐propagating wave of cellular depolarization in the cerebral cortex (Vecchia & Pietrobon, 2012). The neurovascular phenomena propagate within the occipital cortex, with a velocity compatible with the symptoms reported by single patients (Hadjikhani et al., 2001). The initiation of CSD is caused by the localized extracellular elevation of H^+^, K^+^, and other agents, including arachidonic acid and nitric oxide (Pietrobon & Striessnig, 2003). Subsequent to the elevation of these agents to a critical threshold (eg, in the case of K^+^, the threshold is 10–12 nM), a self‐propagating CSD wave is initiated, and it gradually advances across the cortex with a low velocity of 3–5 mm/min, starting from the occipital regions toward the frontal ones (Charles & Brennan, 2009; Pietrobon & Striessnig, 2003). However, there are outstanding questions regarding the association between CSD propagation modality and clinical symptoms in migraine patients. During typical aura, visual symptoms could be associated or followed by sensory and speech disturbances, confirming the forward propagation of CSD wavefront and the involvement of anterior cortical regions, that may become eloquent in single cases. In fact, the majority of migraine patients experience only visual scotoma, so several factors could potentially limit the progression of CSD wavefront, or reduce the possibility for cortical areas other than the visual one to become eloquent during the depolarization phenomenon. Functional and anatomical characteristics of cortical regions, especially in the occipital area, could explain the facilitation of CSD progression and the associated symptoms perception in migraine with aura (Gaist, et al, 2018; de Tommaso et al., 2017;). In this multifaceted scenario, the complex and highly individual characteristics of the brain cortex suggest that the geometry might have a significant impact in supporting or contrasting the propagation of cortical spreading depression. In recent studies, CSD propagation was studied by using a computational neuronal model distributed throughout a realistic cortical mesh, integrated with patient‐specific diffusivity tensors derived locally from diffusion tensor imaging (Kroos, Diez, Cortes, Stramaglia, & Gerardo‐Giorda, 2016; Kroos et al., 2017). These theory and single cases applications suggested that cortical geometry could explain some features of CSD propagation: In the present study, we aimed to apply this computational neuronal model to single cases of migraine with aura.

## 2 MATERIALS AND METHODS

### 2.1 Clinical dataset

The dataset corresponds to four female and one male subjects, with an age of 27.4 ± 9 (range 21–43) years, suffering from typical migraine with aura (cod). Three patients presented with associated migraine without aura (Table 1). These patients were selected at the Applied Neurophysiology and Pain Unit of Bari Policlinico General Hospital and diagnosed in accord to the recent clinical criteria (2018). Patients were requested to pay attention and write down in detail a description of the aura symptoms experienced during the first migraine with aura attack occurring after the first visit, as well as their temporal evolution. Preventative treatment for migraine was initiated after the occurrence of the migraine with aura episode reported in the present study (Table 1). The MRI acquisition was performed in the SS. Annunziata Hospital, Taranto, Italy. The study was approved by the local ethics committee of Bari Policlinico General Hospital. Patients were informed about the aims and methodology of the study and signed an informed consent. The patient data consisted of a T1‐weighted image serving as a basis for the cortical reconstructions, and a diffusion‐weighted image (DWI) providing among other things the apparent diffusion coefficient (ADC). Differences in the tissue structure reflect into differences in the diffusion coefficient, and the ADC values describe the underlying tissue structure by the average of diffusion of water molecules in the three principal directions in a voxel.

**Table 1:** Main clinical features and details of the migraine aura in five patients. In the last column, the propagation time of the simulated CSD in each case is reported

Pz	Age	Age of illness (years)	Migraine with aura frequency (days with migraine/month)	Migraine witout aura frequency (days with migraine/month)	Suggested preventive treatment	Diagnosis	Symptoms characteristics and duration	Hemisphere	Propagation time (min)
1	24	9	5		Topiramate	Migraine with aura (typical)	Luminous scotoma in central visual field 0–2 min Right hemifield scotoma 2–10 min Right and left paresthesias 10–40 min Aphasia, aprassia 10–60 min Migraine headache (60 min)	Left Right	16.23 16.13
2	22	10	1		None	Migraine aura (typical) with non migraine headache	Luminous scotoma in central visual field (0–5 min) Right hemifield scotoma (5–15 min) Right arm motor symptoms, right arm sensory symptoms. aphasia 15–60 min) (non migraine headache −40 min)	Left Right	15.72 15.44
3	22	10	0.5	0.5	None	Migraine with aura (typical) *plus* Migraine without aura	Luminous scotoma in right and left visual fiels (1–2 min) Right facial and brachial sensory symptoms (15–60 min) Aphasia (60–120 min) Migraine headache (60 min)	Left Right	16.25 16.23
4	42	25	2	2	Topiramate	Migraine with aura (typical) *plus* Migraine without aura	Central luminous scotoma (0–30 min) Right facial and brachial parestesias (15–60) Aphasia (30–80) Migraine headache (60 min)	Left Right	14.1 14.3
5	27	15	0,5	7	Flunarizine	Migraine with aura (typical) *plus* Migraine without aura	Black scotoma in full visual field 1 min Luminous scotoma in peripheral left visual hemifield: 1–3 min Migraine headache (5 min)	Left Right	14.51 14.13

### 2.2 Data acquisition

MRI data were acquired with a Siemens Magnetom Aera 1.5 T scanner using a T1‐weighted 3D sequence with the following parameters: TR = 2,650 ms, TE = 106 ms; flip angle = 10°; parallel imaging (mSENSE) acceleration factor = 1.5; acquisition matrix size = 256 × 256; FoV = 260 × 260 mm and slice thickness = 1.1 mm, and 176 contiguous sections. The DWI image was acquired with the same scanner using a diffusion‐weighted 2D sequence with the following parameters: TR = 2,650 ms, TE = 106 ms; flip angle = 90°; acquisition matrix size = 192 × 192; FoV = 229 × 229 mm and slice thickness = 5 mm, three diffusion directions, and 23 contiguous sections.

### 2.3 Preprocessing

In order to simulate personalized CSD propagation, we solve a reaction‐diffusion equation describing the extracellular potassium dynamics on a triangulated cortical mesh reconstructed from the patient's T1‐weighted image. Additional personalization is included by using diffusivity tensors derived locally from the patient's DWI data. In this section, we describe the preprocessing steps required to reconstruct the geometry from the T1 data and register the diffusion coefficients obtained from the DWI data with this geometry.

The cortical geometry was obtained from the high‐resolution anatomical T1 with the FreeSurfer (http://surfer.nmr.mgh.harvard.edu/) image analysis suit: for further details, see (Kroos et al., 2016, 2017) and references therein. The brain reconstruction process was run with the FreeSurfer version 5.3.0 on BCAM's in‐house cluster Hipatia featuring 18 nodes (1 with Nvidia Tesla K40 GPU) for 624 cores with 4TB RAM and Infiniband network connectivity using CentOS 7 64 bits. For the sake of comparability, all reconstructions were run on the same node of the cluster with 32 cores (2xProcessor Intel(R) Xeon(R) CPU E5‐2683 v4 @ 2.10 GHz with 16 cores) and 256 GB RAM. The brain reconstruction with FreeSurfer results in a triangulated mesh of the cortical surface where neuroanatomical labels are assigned to each mesh point. This way, each point of the triangulated mesh is associated to a specific region of the Brodmann atlas (Brodmann, 2006).

The DWI data were processed with the FSL software. (http://fsl.fmrib.ox.ac.uk/) First, an eddy current correction was applied to fix the changes produced by the variations in gradient field directions during the acquisition and to overcome the artefacts produced by head motion. The T1 and DWI brain data were extracted with a brain extraction tool (BET) using a fractional intensity threshold of 0.3 and a local fitting of the DWI data was then applied to compute the tensor model at each voxel. To project this data onto the mesh obtained with FreeSurfer from the T1 image, a linear transformation was first computed between the T1 image and the ADC map using 6 degrees of freedom and taking the mutual information as the cost function. Then, the transformation from T1 native space to the FreeSurfer structure space was computed with the FreeSurfer tkregister2 function. Combining these transformation matrices and applying them to the ADC data, we obtain these values in the FreeSurfer space. Finally, we used the FreeSurfer function mri_vol2surf to project the diffusion data onto the brain mesh with a projection fraction of 0.5 to sample in the middle of the cortical surface and prevent bias from the white matter or the extracerebral fluid.

**Figure 1:**
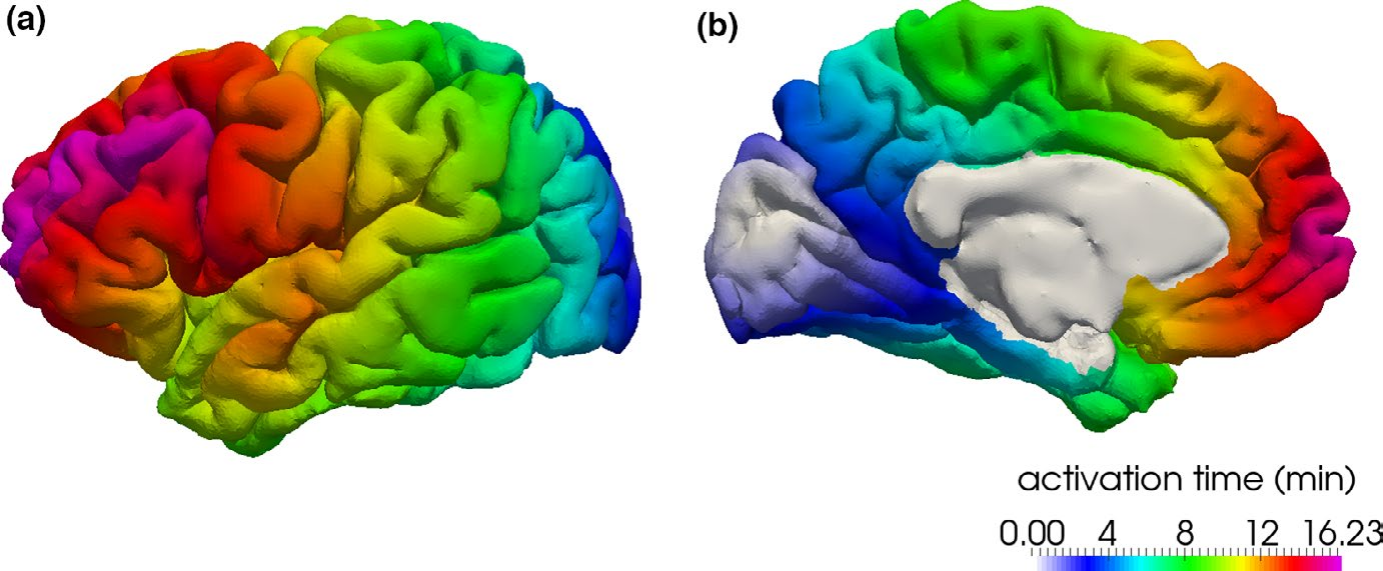
(a) The smoothed mesh reconstructed from T1‐weighted image for the left hemisphere of patient 1. (b) The ADC values on the surface mesh after taking out the medial wall (bottom) for the left hemisphere of patient 1

This process provides, for each patient, a brain geometry in the form of a triangulated mesh with personalized ADC values associated to each grid point. A couple of further steps were needed for such geometry to be properly used in the CSD simulation. As a first step, the mesh was smoothened to eliminate artifacts and reduce the noise: The Taubin algorithm with equal weights was used up to a volume loss of 5% choosing the parameters *λ* = 0.33 and *μ* = 0.34 (Taubin, 1995). As a second step, the medial wall was eliminated from the geometry for the CSD simulation to prevent a speed‐up of the CSD wave that would naturally not take place. An example of the resulting smooth mesh and the ADC values on this smooth mesh is shown in Figure 1 for left hemisphere of subject 1.

### 2.4 CSD simulation

In order to simulate CSD, we model the wave of extracellular potassium surge associated to the CSD propagation with a reaction‐diffusion model, first introduced in (Kroos et al., 2016) by some of the authors of this study. This model is approximated in time by finite differences and in space by finite elements. For each subject, the model equations include the patient‐specific diffusivities derived from the subject DWI data and are solved on the cortical geometry reconstructed from individual MRI scans. We refer the reader to the Appendix S1 for a summary of the computational model, and to (Kroos et al., 2017) for the full description. We normalize these ADC values by the overall mean and use the same model parameter values as in (Kroos et al., 2017).

The Regions Of Interest (ROI) for this study are identified by means of the most essential Brodmann areas—BA—(Brodmann, 2006) that include the primary somatosensory cortex (BA 1–3), the primary motor cortex (BA 4), Broca's area (BA 44, BA 45), and the visual cortex (V1, V2 or BA 17, BA 18, respectively). For each subject, the CSD wave is initiated in the Brodmann area where the first symptoms were recorded, usually the (primary) visual cortex V1 and propagates across the whole cortex.

## 3 RESULTS

**Figure 2:**
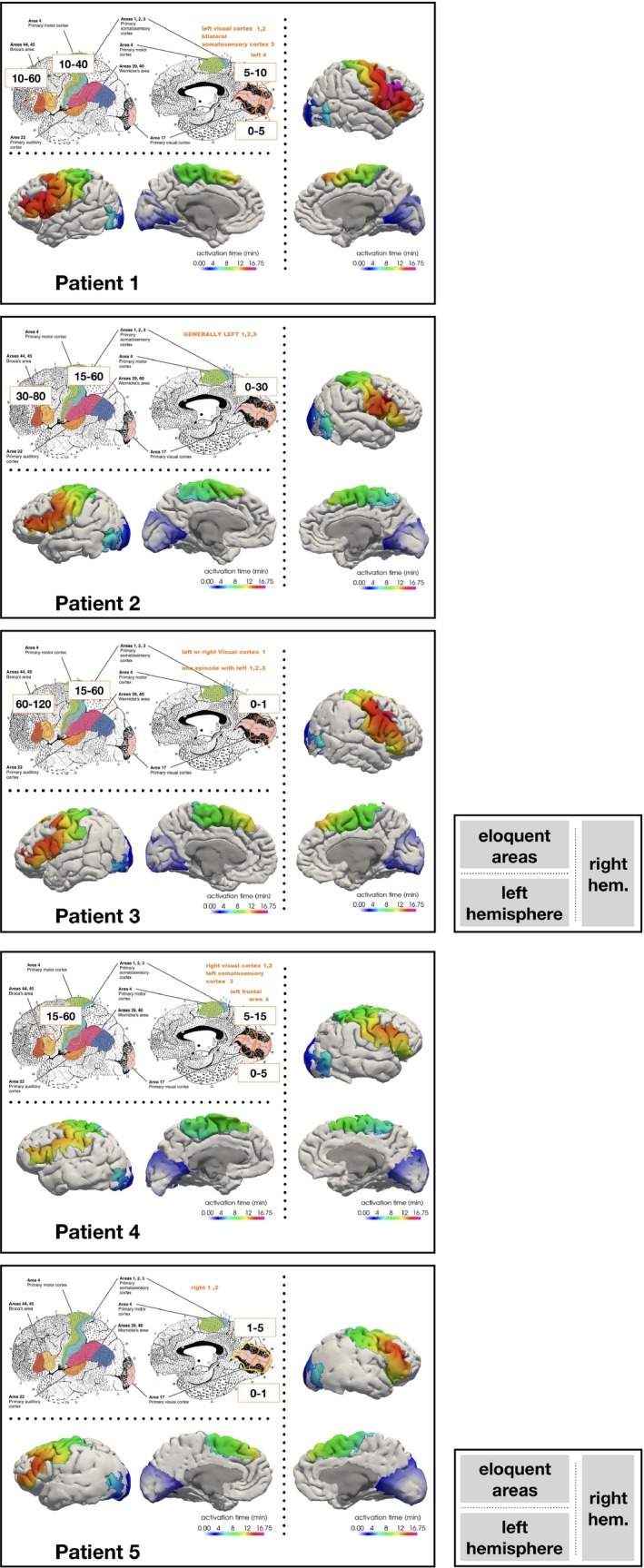
The arrival time (activation time) of the CSD wave in the left (left side) and right (right side) hemisphere of patients 1–5. At the top, the Brodmann areas maps and the temporal evolution of aura symptoms are described, in accord with the patients' reports (Table 1)

Clinical details and characteristics of described aura symptoms are reported in Table 1. For all subjects, we simulated the CSD propagation across the whole cortex and recorded the arrival time of the wavefront in every point of the mesh. In Figure 2, we show the arrival times of the K^+^ wave in the Brodmann areas associated with the clinical symptoms reported by the patients. In Figure 2 (referring to patient 1), the regions involved in the migraine attack (in the specific: BA 1–4, 17/18, 44/45) are colored according to the arrival time of the CSD wavefront. We can observe that the wave starting in the visual cortex reaches the regions that become eloquent during the migraine attack in a sequence that matches the onset of the reported symptoms. From the visual cortex, the wave propagates to the bilateral somatosensory cortex (BA 1–3) and Broca's area (BA 44/45), in agreement with the patient report (Table 1). The times of symptoms appearance and duration are coherent with the speed to CSD wave described in the model. The CSD propagations for the other four patients are shown in Figure 2. In patient 2, starting from the left visual cortex, the CSD waveform reached the bilateral somatosensory cortex and Broca area (Figure 2), though patient described symptoms attributable to the left hemisphere, similarly to patient 3 and 4 (Figure 2). Patient 3 reported initial visual symptoms attributable to both hemispheres, followed by a prevalent involvement of the left somatosensory and Broca areas (Figure 2). In the left hemisphere of patient 4, BA 1–4 and 44 were reached by the simulated CSD wave almost simultaneously, which corresponded to the concomitant sensory and speech disturbances. In patient 5, the model showed a CSD progression toward anterior regions, despite the patient described visual symptoms, referring to the right BA 17 and 18 (Figure 2).

For a better overview, we list the maximum time the wave takes to spread across the whole cortex, starting in the V1 for all migraine patients in Table 1.

## 4 DISCUSSION

In this single case study, we simulated the spreading of cortical depression on a realistic model derived from MRI and DWI data of five migraine with aura patients. According to (Kroos et al., 2017), the velocity of wave propagation in the whole cerebral cortex was around 15 min in all cases. The temporal and topographic evolution of clinical symptoms was in accord with a starting location from V1 and V2 in three cases, and V1 in two cases, with an involvement of sensory regions corresponding to facial and brachial representation and subsequent involvement of Broca's area in all but one patient, who simply reported visual stimuli. Many considerations could rise from these simulations. The geometric configuration of migraine patient cortexes supports in principle a bilateral spreading of CSD wave, with a possible symmetric and quite contemporary involvement of right and left hemispheres. This potential bilateral behavior is in accord with the animal models of CSD (Chang et al., 2010). Few studies dealt with brain electrical changes during migraine aura, as the rare EEG recordings during the aura phase, did not detect the CSD phenomenon (de Tommaso, 2019). In fact, spread of electroencephalographic slow potential changes could not be evident, presumably due to a superposition of volume‐conducted electroencephalographic signals from widespread cortical generators (Drenckhahn et al., 2012).

One MEG study explored spontaneous and induced migraine auras in migraine patients and controls (Bowyer, Aurora, Moran, Tepley & Welch, 2001). Authors observed a DC shift of about 120 s duration, which involved unilateral occipital regions in spontaneous visual aura and bilateral occipito‐temporal‐parietal regions in induced aura. In this second case, the temporo‐parietal regions, where the DC was recorded, were clinically silent, as the patients experienced only visual symptoms. According to these results, the bioelectrical depolarization phenomenon could not be sufficient to trigger aura symptoms perception. In a pivotal FMRI study, the visual stimuli perception corresponded to the unilateral occipital cortex changes of bold signal (Hadjikhani et al., 2001). In that study, a focal and strictly unilateral increase in brain oxygen level‐dependent (BOLD) signal (i.e., hyperemia) in occipital cortex was observed, followed by a longer‐lasting decrease during which functional coupling was impaired, mimicking the known cerebral hemodynamic effects of SD. The BOLD transient propagated within the cortex at a rate of approximately 3.5 mm/minute and was retinotopically congruent with the patient's visual perception. In a more recent, FMRI study conducted in single cases of migraine with aura patients experiencing bilateral visual stimuli, bold signal changes were detected in both hemispheres (Arngrim et al., 2017). The FMRI data outline that the perfusion changes are congruent to the clinical data, more than the bioelectrical phenomenon. Following this argument, the depolarization phenomenon could become clinically eloquent, if the vascular and perfusion changes occur, a factor probably dependent upon the dynamical situation of the cerebral circulation, as the neurovascular coupling is a mutable phenomenon (Charles & Brennan, 2009). The entity of CSD phenomenon could thus change among cortical areas, for different concentration of extracellular K ions and consequent activation of vascular phenomenon.

According to this CSD progression model, the extracellular K^+^ propagation could involve large cortical regions, with high probability of depolarization of nociceptive afferents and induction of headache (Charles & Brennan, 2009; Karatas et al., 2013; Pietrobon & Moskowitz, 2014). In all patients, migraine or not migraine headache started after the initiation of aura symptoms, being often in contemporaneity with them. We can suppose that if the geometry of migraine brain supports a massive phenomenon of K^+^ extravasation across the cortex, migraine could occur when a sufficient number of meningeal and vascular trigeminal nociceptive afferents could be activated during the CSD progression (Brennan & Pietrobon, 2018).

In our CSD propagation simulation, the depolarization wave reached in all cases the primary and secondary somatosensory cortex, especially in the areas topographically related to the trigeminal sensory innervation, and the anterior cingulate cortex, all involved in pain processing (Legrain, Iannetti, Plaghki, & Mouraux, 2011; Tracey & Mantyh, 2007). The functional modification of such cortical areas, although transitory and reversible, could possibly favor headache persistence through defective descending control. (Brennan & Pietrobon, 2018).

The realistic models of CSD progression, preliminarily proposed in this study, suggest that the cortical geometry of migraine patients could subtend a diffuse and massive bioelectrical phenomenon. The simulated CSD was in fact correlated only in part with clinical manifestation of aura, which in 4 of the presented cases were attributable to unilateral hemispheric involvement. Three out of five patients presented with both migraine with and without aura attacks, so it is conceivable that the same depolarization phenomenon could induce migraine headache, independent from prodromal symptoms (Ayata, 2010). At this stage, we have no data to support the hypothesis that the aura symptoms perception could happen in relation to phenomena other than CSD progression, as local vascular changes (Hadjikhani et al., 2001; Brennan & Charles, 2010). We can suggest that the geometry of migraine brain could subtend a large and bilateral depolarization wavefront starting from the occipital regions toward cortical multifunctional regions, possibly capable to activate pain afferents devoted to the processing of trigeminal nociceptors signals. In addition, we observed a possible dissociation between the CSD progression and the perception of aura symptoms.

Further studies will include normal geometrical models, as well as models of patients with chronic migraine, to understand if migraine brain has a basal anatomical configuration able to facilitate CSD phenomena, which could, in turn, modify functional properties of brain regions devoted to pain modulation and persistence.

## CONFLICT OF INTEREST

The authors declare no competing interest.

## AUTHOR CONTRIBUTIONS

MdT, SS and, LGG designed the study; LGG, JMK, and SS developed the model; JMK implemented the computational model, processed the data and the results; MdT and EV recruited the patients and collected clinical data; NB performed the patients imaging acquisition; and JMK, LGG, and MdT wrote the manuscript. All authors revised the manuscript.

## Data Availability

The data that support the findings of this study are available from the corresponding author upon reasonable request.
