# Supplementary material for: Clinical correlates of mathematical modeling of cortical spreading depression: Single‐cases study
Source: Brain Behav. 2019 Sep 10;9(10):e01387. doi: 10.1002/brb3.1387 (PMC6790336; doi:10.1002/brb3.1387)
Supplement: Supplementary file 1 [file BRB3-9-e01387-s001.docx]

**Supplementary Material**

**Mathematical model for CSD wave propagation**

In accordance with the potassium hypothesis (Zandt et al, 2015), we model the propagation of the extracellular potassium, whose concentration we denote by *k*, as the driving force of CSD propagation. This model can be extended to reproduce the typical electrophysiological behaviour during a spreading depression (Gerardo-Giorda and Kroos, 2017). For the sake of simplicity, we focus on the potassium propagation. We describe the temporal evolution and propagation of the extracellular potassium *k* with a coupled reaction-diffusion differential model as

$\begin{matrix} \frac{\partial k}{\partial t} & = & \text{div}_{\Sigma}(D\nabla_{\Sigma}k)-F(k,w), \\ F(k,w) & = & \eta_{1}(k-k_{0})(\begin{aligned} 1-\frac{k}{k_{th}} \end{aligned})(\begin{aligned} 1-\frac{k}{k_{p}} \end{aligned})-\eta_{2}(k-k_{0})w, \\ \frac{\partial w}{\partial t} & = & \eta_{3}(k-k_{0}-\eta_{4}w), \end{matrix}$ (1)

where *D* is the diffusion tensor, *w* is a recovery variable, $\eta_{1,}\eta_{2,}\eta_{3}$and $\eta_{4}$are parameters, while $k_{0},k_{th},k_{p}$are the resting, threshold, and peak values of the potassium concentration *k*, respectively. The values of these parameters are reported in Table S.1. The computational domain is a 2D surface $\Omega\subset\mathbb{R}^{3}$reconstructed from T1 imaging, and the space-differential operators in equation (1) are the tangential counterparts of the classical divergence and gradient operators. Problem (1) is defined on a computational domain $\Omega\times(0,T)$and its mathematical well-posedness is obtained by imposing initial conditions $k^{0}(x)=k(x,0)$and $w^{0}(x)=w(x,0)$in $\Omega$and suitable boundary conditions on $\partial\Omega$.

To apply the CSD model to the real brain geometries of migraine patients obtained from MRI imaging, we consider an isotropic diffusion tensor, $D=\delta I_{2}$where $\delta$is the ADC value derived from the DWI data and $I_{2}\subset\mathbb{R}^{2}$is the identity. We initialise the problem by imposing *k* = 64 mM in the region of first symptoms and let the wave propagate on the whole cortex.

| Parameter | Description | Value | Unit |
| --- | --- | --- | --- |
| $k_{0}$ | Resting value of potassium concentration | 4 | mM |
| $k_{th}$ | Threshold value | 11.8 | mM |
| $k_{p}$ | Peak value of potassium concentration | 64 | mM |
| $\eta_{1}$ |  | 0.2667 | s^-1^ |
| $\eta_{2}$ |  | 0.4806 | s^-1^ |
| $\eta_{3}$ |  | 3.333e-5 | s^-1^ |
| $\eta_{4}$ |  | 60 | mM |

**Table S.1:** Model parameters for the propagation of the potassium concentration.

**Numerical Approximation**

We discretise problem (1) by finite elements in space and finite differences in time. Let *t^n^ = nΔt* for *n = 0,…,N*, be a discretisation of the time interval *(0,T)*: we denote with *k^n^* and *w^n^* the approximation of the model values of *k* and *w* at time *t^n^*. Using an implicit-explicit (IMEX) scheme to advance from time *t^n^* to *t^n+1^*, the recovery variable *w^n+1^* can be solved explicitly on each interval [*t^n^,* *t^n+1^*], and is used in the expression *F* to compute *k^n+1^*. The overall scheme reads:

**update:** $w^{n+1}=\frac{k^{n}-k_{0}}{\eta_{4}}+(\begin{aligned} w^{n}-\frac{k^{n}-k_{0}}{\eta_{4}} \end{aligned})\text{exp}(-\eta_{3}\eta_{4}\Delta t)$

**update:** $F^{n+1}=F(k^{n},w^{n+1})$

**solve:** $(M+\Delta tS)k^{n+1}={Mk}^{n}-\Delta tMF^{n+1}$

where *M* and *S* are the classic finite element mass and stiffness matrices. For further details about the numerical implementation we refer to (Kroos et al, 2017).

**Simulation**

The numerical simulations of the CSD model were performed with a self-developed code in Matlab (MathWorks Inc., Natick, MA), choosing a constant time step $\Delta t=0.6$s. The IMEX scheme described above requires to solve, at each time step, a linear system associated with a linear parabolic system. The linear systems are solved by a preconditioned conjugate gradient (PCG) method (Saad, 2003). In our simulations, we used as a preconditioner an incomplete Cholesky factorization of the matrix $M+\Delta tS$, with pivoting and drop tolerance set at 10^−6^. Choosing the solution at the previous time step as initial guess for the PCG, combined with the low drop tolerance in the preconditioner and the slow propagation of the CSD wave ensures that one PCG iteration per time step is sufficient to recover the solution.

In Figure 1 we show the arrival times of the CSD wave in the left hemisphere of patient 1.

**Bibliography**

1. Gerardo-Giorda L, Kroos JM (2017). Multiscale modeling and simulation of cortical spreading depression propagation, *Computers and Mathematics with Applications,* 74.5, pp. 1076–1090.
2. Saad Y (2003). Iterative methods for sparse linear systems. 2nd ed.
3. Zandt B, ten Haken B, van Putten MJAM (2013). Diffusing substances during spreading depolarisation: Analytic expression for the propagation speed, triggering and concentration time courses. *Journal of Neuroscience,* 33.14, pp. 5915–5923.
